# The pitfalls of untested assumptions and unwarranted/oversimplistic interpretation of cultural phenomenon: a commentary on Sajjadi et al. (2023)

**DOI:** 10.3389/fpsyg.2023.1248246

**Published:** 2023-09-07

**Authors:** Mojtaba Elhami Athar

**Affiliations:** Darkmind Research Group, Tabriz, Iran

**Keywords:** psychopathy, cross-cultural, middle-east, factor structure, CAPP-SR

## Introduction

*Amicus Plato, sed magis amica veritas*.

*Plato is dear to me, but dearer still is truth*.

—Aristotle 384–322 B.C.

Cross-cultural studies showed interesting differences in psychopathy construct between Western and Eastern cultures (e.g., Neumann et al., 2012), suggesting that the factor structure of psychopathy measures may vary across cultures. Therefore, it is important to first investigate the factor structure of psychopathy measures in diverse cultures to avoid introducing confounding findings.

Sajjadi et al. (2023) recently published a study that aimed to validate the Persian version of the Comprehensive Assessment of Psychopathic Personality-Self-Report (CAPP-SR). In my view, this study has significant methodological/conceptual problems that could undermine the interpretations/utility of the results. While acknowledging their contribution, I respectfully highlight specific concerns regarding the absence of a factor structure test, the validity of external measures, and unwarranted/oversimplified interpretations of cultural phenomena. These issues require attention and are discussed in detail below.

### Lack of factorial structure test

Factor analysis is a type of latent variable modeling technique that condenses observed variables into underlying factors, capturing shared variation and grouping them based on stronger covariation. In personality disorders research, it is used to validate assessment tools and uncover the structure of related traits or symptoms (Watts et al., 2023). A good factor structure enhances the validity, reliability, and interpretability of a measurement instrument, while a poor factor structure can lead to issues of invalidity, unreliability, and lack of interpretability (e.g., Hoben et al., 2016; Boateng et al., 2018). Prior studies showed that the factor structure of psychopathy measures developed in Western cultures often required modifications to achieve a satisfactory model fit in Iran (e.g., Shariat et al., 2010; Ebrahimi et al., 2021, 2022; Elhami Athar et al., 2023b). Regarding CAPP-SR, Shou et al. (2021) showed that certain items of the CAPP-SR did not yield sufficient psychometric information in a Chinese sample, and a majority of the symptom scales (22 out of 33) exhibited low internal consistency and test information. Shou et al. (2021) suggested that ratings of some CAPP-SR items might be influenced by cultural norms and social restrictions in China.

Despite this substantial evidence, Sajjadi et al. (2023) did not examine the factor structure of the Persian CAPP-SR which raises doubts about the reliability and interpretation of their findings. Previously, Shariat et al. (2010) showed that PCL:SV items that assess Superficial, Deceitful, and Grandiosity traits did not function well in Iran maybe due to *ta'arof* —the Iranian national trait of exaggerated politesse, modesty, and self-deprecation (Shariat et al., 2010). Possibly, *ta'arof* might explain the elimination of some items from the Persian Antisocial Process Screening Device (APSD; Ebrahimi et al., 2021), the Youth Psychopathic Traits Inventory-Short (YPI-S; Ebrahimi et al., 2022), and the Proposed Specifiers for Conduct Disorder scale (PSCD; Elhami Athar et al., 2023a,b). Therefore, if we suppose that *ta'arof* influences the functioning of some items in psychopathy measures, it is expected that the originally proposed factor structure model of the CAPP-SR would have yielded acceptable model fit in the Iranian sample after eliminating some items the CAPP-SR symptom scales of Self-Aggrandizing (items 22 “*I have special qualities,”* 41 “*I am (or, will one day be) very famous,”* and 50 “*I am a very important person.”*) and Sense of Entitlement (items 38 “*I often find that I have to be quite assertive in getting what I deserve,”* 64 “*I deserve special treatment,”* and 66 “*I might be perceived as demanding, but I am also deserving”*).

Also, the CAPP-SR item 38 (“*I often find that I have to be quite assertive in getting what I deserve,”*) has diverse implications for Iranians depending on the context. In interpersonal relationships, assertiveness contradicts cultural norms of humility/modesty, potentially being perceived as disruptive/arrogant. Additionally, indirect communication is favored in Iranian culture. Iranians often rely on relationships/personal connections to negotiate and achieve objectives. Meanwhile, Iranians believe that being assertive, demanding, and persistent is important when navigating bureaucratic processes. Specifically, many Iranian organizations/agencies experience significant inefficiencies, leading to challenges in completing paperwork promptly.^1^ Iranians often depend on building relationships and leveraging personal connections to expedite these bureaucratic processes. If this approach proves ineffective, individuals adopt an assertive behavioral style which is considered a normal and even advantageous quality among Iranians when dealing with bureaucracy. Meanwhile, the CAPP-SR item 38 does not fully capture the cultural implications of assertiveness in Iran. Additionally, items of the Self-Centered symptom scale, such as items 26 (“*I am mostly just interested in things that apply to me”*) and 57 (“*To be honest, I don't really care about other people's opinions”*), are in stark contrast with the predominantly collectivistic culture of Iran and may not function well in Iran.

Relatedly, Iran is a diverse country with multiple ethnic/linguistic groups, each having its own distinct language, traditions, and folklore (for a review see Elhami Athar et al., 2021) which could influence the manifestation and prevalence of psychopathic traits. In this vein, the study of Sajjadi et al. (2023) included a combined sample of university students from Tehran and Ahvaz. Ahvaz, located in Khuzestan province differs significantly from Tehran province in terms of socio-economic status (SES) and includes a substantial number of individuals with Arabic as their mother tongue. These cultural differences could potentially influence ratings of psychopathy measures. Lastly, in certain Eastern countries, including Iran, a division between “us” and “them” exists, which can normalize the expression of psychopathic traits toward a certain group. This division, observed in recent protests in Iran, may impact individuals' perception/response to specific items in psychopathy measures (for more information, see Elhami Athar, 2023). Indeed, research is crucial to examine the factor structure and item functioning of the CAPP-SR in Iranian culture. By conducting such studies, researchers can explore whether the existing factor structure of the CAPP-SR aligns with the cultural context of Iran or if modifications and adaptations are necessary to capture the specific cultural nuances. It can also shed light on the functioning of individual items within the Iranian cultural context, highlighting any potential issues or discrepancies that may arise due to cultural differences. Such studies would provide valuable insights into the cultural influences on individuals' responses to the CAPP-SR, enhancing our understanding of the psychopathy construct within a specific cultural context.

### Concerns about external measures

The use of appropriate measures for examining the validity of a new measure is crucial for ensuring the accuracy and credibility of research findings. There are significant concerns regarding the external measures employed by Sajjadi et al. (2023). For example, the Persian translation of the Levenson Self-Report Psychopathy Scale (LSRP) has several notable translation errors. For instance, the term “manipulation” does not possess an exact Persian equivalent, and a more appropriate translation could be “fooling.” Unfortunately, this issue was not addressed in the translation of the corresponding LSRP item (“*I enjoy manipulating other people's feelings”*) or the CAPP-SR item 96 (“*Sometimes it is frankly necessary to manipulate others to achieve something”*). Similarly, in the LSRP item “*Cheating is not justified because it is unfair to others,”* the translation inaccurately rendered “cheating” as “betrayal,” deviating from the original intent. It remains unclear whether Sajjadi et al. (2023) addressed these translation errors before administering the LSRP. Similarly, four items were removed from the factor structure of the Persian Inventory of Callousness-Unemotional Traits (ICU) in the study referred to by Sajjadi et al. (2023). While the removal of items 2 and 10 aligns with previous studies, the exclusion of items 4 and 13 raises concerns that require attention. Meanwhile, the number of administered and scored ICU items by Sajjadi et al. (2023) remains unclear.

### Oversimplified interpretation of cultural phenomena

Sajjadi et al. (2023) found that the mean score of an item from the Sense of Entitlement symptom scales (“*I often find that I have to be quite assertive in getting what I deserve”*) tended to be elevated. While they acknowledged that assertiveness is a complex concept, their assertion that “*among Iranians, assertiveness is perceived as a valued and adaptive behavior associated with healthy personality adjustment (p.15)”* does not fully align with Iranian culture. I previously discussed about cultural implications of assertiveness in Iran. Expanding on the previous discussion, it is worth noting that assertiveness holds significant value in academic bureaucratic procedures in Iran, which may come as a surprise to non-Iranian readers. Iranian students must be assertive, demanding, and persistent to obtain timely approvals for their academic requirements, which could explain the elevated CAPP-SR item 38 level in Sajjadi et al.'s (2023) study which focused on university students. Overall, assertiveness in Iranian culture is nuanced, and context-dependent.

Additionally, Sajjadi et al. (2023) found that the mean score for the Unreliable symptom scale was significantly higher and was not significantly associated with other psychopathy scales. They found this partially consistent with findings that Superficial, Deceitful, and Grandiose items of PCL-SV did not effectively differentiate Iranian participants with psychopathy from those without (Shariat et al., 2010). However, they do not explain how the findings from these two studies are partially in line. In addition, Sajjadi et al. (2023) cited findings from two national surveys indicating that a majority of Iranians perceived an increase in traits and behaviors related to hypocrisy, insincerity, and flattery within their society. They reported that these findings align with their observed pattern of results related to the Unreliable symptom scale. It is worth noting that these national surveys were conducted in 2002 and 2011, which are not reliable currently, given the significant political, economic, and social changes in Iran in recent years. Sajjadi et al. (2023) also claim that the Iranian culture of *ta'arof* would explain the pattern of findings on the Unreliable symptom scale, but unfortunately, they provide mixed definitions and implications for *ta'arof*. For example, they cited references indicating that *ta'arof* can be viewed as “*a form of deception, manipulativeness, and insincerity …. false promise based on a false premise (p.15)”* while also explaining that an Iranian communicator knows the rules of *ta'arof* and is able to understand that if, for example, someone makes promises, that can be merely following cultural norms and that does not mean that the speaker will keep those promises. In my perspective, the utilization of *ta'arof* in interpreting findings from psychopathy measures should be approached with caution, given the absence of established empirical measures to assess *ta'arof* and examine its correlates.

Sajjadi et al. (2023) further cited a reference suggesting that Iranians approve hypocrisy and flattery and view them as facilitators and shortcuts to success. However, there are important considerations to address here. Importantly, while hypocrisy and flattery can be associated with success when utilized skillfully (which is the case with successful psychopaths), the cross-cultural implications of Iranians' approval of hypocrisy/flattery remain unclear, and comparative studies are needed to understand how individuals from other cultures perceive these behaviors. Using such texts without proper context or supporting evidence can indeed lead to the misrepresentation of a cultural group. It is essential to approach cultural studies with caution and avoid misrepresenting a cultural group.

## Discussion

Research on psychopathy heavily relies on Western cultures which disregards the cultural specificity of this condition. Psychopathy measures were also developed and standardized on Western populations. Understanding cultural variations in psychopathy is crucial for practical and theoretical reasons. Studies are needed to examine psychopathy measures across diverse cultures with exact methodology and unbiased interpretation of cultural phenomena. Finally, as suggested by an anonymous reviewer, journal editorial teams could contemplate the inclusion of reviewers who are well-versed in the relevant cultural context, such as *ta'arof*, when dealing with manuscripts that hold significant cultural implications.

## Author contributions

The author confirms being the sole contributor of this work and has approved it for publication.

## Footnotes

^1^This can be partly attributed to the lack of a scientific approach in the recruitment and selection of human resources, where relevant qualifications and attributes are not given sufficient consideration. However, a comprehensive discussion of this topic is beyond the scope of this article.
